# Establishing a preliminary normative database of oral efficiency for children: the Test of Masticating and Swallowing Solids Application (ToMaSSApp)

**DOI:** 10.1007/s00431-026-07110-2

**Published:** 2026-06-03

**Authors:** Anna G. Willocks, Ksenia M. Bykova, Elena Moltchanova

**Affiliations:** 1 Rose Centre for Stroke Recovery and Research, School of Psychology, Speech and Hearing, University of Canterbury, 249 Papanui Rd, 8014 Christchurch, New Zealand; 2 School of Mathematics and Statistics, University of Canterbury, Private Bag 4800, Christchurch, 8140 New Zealand

**Keywords:** Swallowing, Child, Adolescent, Mastication, Deglutition, Dysphagia

## Abstract

The Test of Masticating and Swallowing Solids (ToMaSS) is a diagnostic tool that measures oropharyngeal efficiency of solid bolus consumption through the number of bites, mastication cycles, swallows, and time taken. An application (ToMaSSApp™) has been used to administer the assessment. The project aimed to establish a preliminary normative database for the ToMaSS-C in children administered with the ToMaSSApp™ and to make comparisons to previously manually collected data. A total of 114 healthy children (57 boys and 57 girls) aged 4 to 19 years (*M* = 9 years, *SD* = 4.6 years) were asked to consume a cracker. The efficiency measures were obtained via the ToMaSSApp™. A generalized linear regression model was fitted for each measure and used to obtain normative tables from the data. Equivalence testing was used to compare the data collected via ToMaSSApp™ to the older manually collected data. A normative dataset for the ToMaSS-C in children was established based on the data collected using the ToMaSSApp™. Within the normative dataset, age affected boys and girls similarly and had a statistically significant effect on ToMaSS-C measures (*p* < 0.05). No equivalence was found between the historical manual and App-based test administration for any ToMaSS-C parameters with larger differences for younger ages.

*Conclusion*: The ToMaSSApp™ is shown to be a feasible and efficient tool in the administration of the ToMaSS-C in the paediatric population. Establishing normative data for the ToMaSS-C is essential for understanding typical processes of oropharyngeal efficiency of solid food consumption as well as identifying and managing dysphagia or oral motor delays.

**Table:** 

**What's Known:** • *ToMaSS can be administered to children (ToMaSS-C) using a manual recording method.* • *The ToMaSSApp*™, *as a digital alternative, has recently been used to establish a normative dataset in adults.* **What is New:** • *The ToMaSSApp*™ *is feasible for paediatric ToMaSS-C administration.* • *A preliminary normative dataset for children was established using the App-based recording method. *

**Supplementary Information:**

The online version contains supplementary material available at 10.1007/s00431-026-07110-2.

## Introduction

Solid consumption is developed in children early in life, beginning in infancy and becoming an essential skill to meet nutritional needs throughout childhood and adulthood [1, 2]. However, for children with oral motor disorders (OMD) who cannot properly chew solids, this skill could be challenging. Such difficulties may be associated with reduced feeding efficiency and nutritional compromise and may also increase the risk of airway invasion [3, 4]. For instance, up to 92% of children with cerebral palsy (CP) experience difficulty consuming solids, and among children with CP and dysphagia, approximately 67% are reported in the literature to be at the airway invasion risk [3]. Therefore, the ability to consume solids should be evaluated and addressed in speech–language therapy sessions as early as possible. Considering that most of the available chewing evaluations are incorporated into broader feeding assessment tools [5], four instruments may be considered for specific evaluation of chewing skills. These are the Mastication Observation and Evaluation Instrument (MOE) [6], Karaduman Chewing Performance Scale (KCPS) [7], the Mixing Ability Test (MAT) [8], and the Test of Masticating and Swallowing Solids (ToMaSS) [9, 10]. However, the MOE [6] primarily provides qualitative observational ratings rather than discrete quantifiable measures that allow the construction of normative data. Similarly, the KCPS [7] is based on clinician-rated performance levels. Whereas the MAT does utilise objective quantitative measures [8], it is not easily accessible due to the required highly specialized software for analysis. At the same time, ToMaSS [9] is an accessible and objective measure of masticatory and swallowing performance, available as a smartphone application ToMaSSApp™, allowing electronic transfer of the data and observation of a patient without distraction of making manual notes.

The ToMaSS test is performed while a patient eats a cracker, and a clinician registers the number of bites, masticatory cycles, swallows, and the duration of ingestion [9]. The test was found to be broadly applicable in the adult population (aged 20 years and older) with dysphagia as a comorbidity of various neurological disorders, such as Parkinson’s disease, Huntington’s disease, stroke, traumatic brain injury, and dementia [9, 11–14]. Considering the significant physiological differences between children and adults regarding typical jaw sensorimotor abilities [15], which could influence the consumption of solids, the ToMaSS-C underwent normative studies in the paediatric population as well. The paediatric ToMaSS-C test, which is conducted while making notes manually, underwent international normative studies in various countries, including India for children aged 6–20 years [16], New Zealand, Germany, Italy, Portugal and the Netherlands for children aged 4–18 years [17]. Both studies demonstrated increasing masticatory efficiency with age. The test was implemented in further studies with healthy children [18], children with Down syndrome, who exhibited slower chewing and prolonged consumption time [19], and children with OMD, 90% of whom showed a deficiency in at least one ToMaSS-C parameter [20]. These five studies [16–20] supported the content and construct validity of ToMaSS-C. In addition, the normative study by Frank et al. and the study involving children with OMD also reported excellent intra-rater reliability [17] and moderate to excellent inter-rater reliability across four ToMaSS-C parameters [17, 20].

ToMaSSApp™ was developed as a digital alternative to the manual recording method used in the ToMaSS test. During ToMaSS-C administration, following the original ToMaSS test protocol [9, 17], the participant consumes a cracker while the examiner records each bite, chew, and swallow in real time by tapping on the touchscreen. The application automatically calculates total test duration and derived ToMaSS parameters, and results can be stored electronically or exported. Normative data were recently established in 437 healthy adults aged 20–99 years using ToMaSSApp™ [21], demonstrating decreased masticatory efficiency with increasing age and identifying significant age and sex effect on ToMaSS parameters in older adults, with the exception of swallows number, thereby supporting the clinical utility of the app. However, the ToMaSSApp™ has not yet been used in a normative study with children, which would allow for its recommendation for clinical use in the paediatric population as well. Moreover, previous normative studies in children and adults overlooked participants aged 18 to 20 years from New Zealand, who should be included and could be added to the paediatric normative dataset in the current study.

The current piloting research project was designed to establish preliminary normative data for the ToMaSS-C test in children aged 4 to 19 years, administered using the ToMaSSApp™, and to compare the dataset with the previously manually collected New Zealand data described in the publication by Frank et al. [14]. It was hypothesised that there would be significant differences between the five age groups for the number of bites, masticatory cycles and total consumption time and that there would be significant differences between genders for the number of bites. It was also expected that there would be no significant differences for the ToMaSS-C data between the normative datasets collected manually (historical data) and using ToMaSSApp™.

## Method

### Participants

Healthy children and young adults aged 4 to 19 years and 11 months were eligible to participate in this current cross-sectional normative pilot study conducted in New Zealand. Healthy status was determined through parent-report or self-report (for participants ≥ 18 years) using a screening checklist included in the consent form. The checklist confirmed the absence of the following exclusion criteria: gluten-related dietary needs, illness at the time of participation, history of previous eating or swallowing difficulties, neurological impairments, any speech, language, or oral motor difficulties or delays. Participants were recruited in person by the researcher (a master’s student in speech and language pathology) at various sporting grounds, by contacting faculties at schools and universities, and through online advertisements. Data were collected by the same researcher at sporting venues and in participants’ homes throughout 2024. No participants withdrew after enrolment. Younger children received a verbal explanation of the study, accompanied by an easy-read information sheet and an assent form, which they signed or marked, alongside parental consent. The participants were assigned to five different age brackets in an attempt to evenly stratify across age and sex (4–5 years, 6–7 years, 8–9 years, 10–13 years, and 14–19 years). These age brackets were similar to previously manually collated normative ToMaSS data described by Frank et al. [17], except for the fifth bracket, which integrated individuals aged 18 to 19 years in the current study. Data collection was terminated when there were at least 20 children in each of the five age groups, considering the gender stratification of at least 10 boys and 10 girls within each age bracket.

### Procedure

The procedure followed was identical to that in Frank et al. [17] and reflected the usual ToMaSS protocol. Children were given one Arnott’s Salada™ Original cracker and instructed to “eat this as quickly as is comfortably possible, and when you’re finished, say your name” [10]. The researcher then observed the child eating the cracker from the side and entered the measures (the number of bites, masticatory cycles, swallows and the consumption time) into the smartphone using the installed ToMaSSApp™ in a non-video-recorded single session.

### Statistical analyses

To generate a normative database, percentiles (2.5th, 5th, 10th, 25th, 50th, 75th, 90th, 95th, and 97.5th) of the predictive distribution were calculated based on the output from a Poisson generalized linear model for the number of bites, chews, and swallows and a log-linear model for the test duration (time) with gender and age as covariates. For the latter, normality and heteroscedasticity of residuals were confirmed using diagnostic plots. Analysis of variance (ANOVA) was then used to determine whether age, gender, and their interaction had a statistically significant effect on the respective output.

Finally, equivalence testing was done to compare bites, chews, swallows, and total mastication time between the app dataset and the historical manually collected normative dataset.

All the analyses were performed in R software [22]. Post hoc pairwise Tukey tests were performed using the emmeans package [23].

## Results

The sample statistics for the ToMaSSApp™ data are shown in Table 1. There were no missing observations.

**Table 1:** Sample statistics for the observed data

Gender	Age, years (mean)	N	Mean (SD) (Range)			
			Bites	Mastication cycles	Swallows	Time (s)
Female	4–5 (4.3)	10	10.1 (5.6) (4–21)	88.7 (27.5) (35–118)	3.8 (1.4) (2–6)	69.2 (16) (38.1–89.1)
	6–7 (6.6)	11	5.5 (1.4) (3–8)	68.9 (19) (47–110)	3.5 (0.8) (2–5)	55.7 (15.3) (43.1–86.2)
	8–9 (8.5)	12	4 (2) (1–8)	56.5 (12.6) (35–81)	3.1 (1.2) (2–5)	43.2 (9.4) (29.4–65)
	10–13 (10.3)	13	3.8 (1.7) (1–7)	48.2 (14.7) (24–76)	2.7 (0.8) (2–4)	40.6 (12) (18.9–67)
	14–19 (18.5)	11	2.3 (0.9) (1–4)	41.7 (9.2) (29–62)	2 (0.4) (1–3)	28.6 (9.1) (18.8–49.8)
Male	4–5 (4.5)	10	6.5 (3.4) (3–13)	80.3 (18.1) (48–104)	3.7 (0.9) (2–5)	60.9 (18.9) (35–97.7)
	6–7 (6.6)	10	4.9 (2) (3–10)	62.5 (20.4) (33–92)	3.7 (0.9) (2–5)	48.3 (11.8) (31–62.7)
	8–9 (8.6)	12	3.5 (2) (1–8)	50.7 (12.4) (37–74)	3.2 (1.3) (2–6)	39.8 (11.8) (28.3–72.2)
	10–13 (10.8)	15	2.7 (1.2) (1–5)	48.9 (11.9) (31–82)	3 (0.7) (2–4)	39.8 (7.5) (31.6–59.1)
	14–19 (17.9)	10	1.2 (0.6) (1–3)	36.7 (8.8) (18–48)	2.7 (1.2) (1–5)	28.1 (9.5) (10–46.6)

**Fig. 1:**
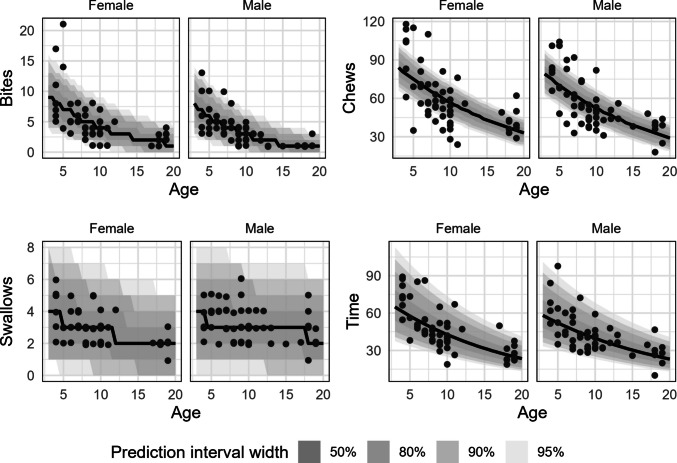
Observed data (black dots), estimated medians (solid lines), and prediction intervals (shaded envelopes) for the ToMaSSApp normative data

The preliminary normative database containing the percentiles of the predictive distribution for each ToMaSS-C parameter by age and gender are shown in Tables 2, 3, 4, and 5. For example, a boy of age 3 is expected to need a median of 8 bites to consume the cracker with the 95% predictive interval of 3 to 14 bites, while a 20-year-old male is expected to need 1 bite with the 95% predictive interval of 0 to 3 bites, respectively. Figure 1 provides a visual representation of the tables with the observed data plotted as black dots, black lines representing the estimated medians, and the progressively lighter envelopes are 80%, 90% and 95% intervals, respectively. The age-specific medians of all four outcomes were found to decrease with age for children of both genders. The somewhat grainy appearance is due to the fact that the response variable is an integer.

**Table 2:** Normative data for the number of bites

Gender	Age	Percentiles (number of bites)								
		2.5	5	10	25	50	75	90	95	97.5
**Male**	**3**	3	3	4	6	8	10	12	13	14
	**4**	2	3	4	5	7	9	10	12	13
	**5**	2	2	3	4	6	8	9	10	11
	**6**	1	2	2	4	5	7	8	9	10
	**7**	1	1	2	3	4	6	7	8	9
	**8**	1	1	2	3	4	5	7	8	8
	**9**	0	1	1	2	3	5	6	7	8
	**10**	0	1	1	2	3	4	5	6	7
	**11**	0	0	1	1	2	4	5	6	6
	**12**	0	0	0	1	2	3	4	5	6
	**13**	0	0	0	1	2	3	4	5	5
	**14**	0	0	0	1	2	3	4	4	5
	**15**	0	0	0	1	1	2	3	4	4
	**16**	0	0	0	0	1	2	3	4	4
	**17**	0	0	0	0	1	2	3	3	4
	**18**	0	0	0	0	1	2	2	3	4
	**19**	0	0	0	0	1	1	2	3	3
	**20**	0	0	0	0	1	1	2	3	3
**Female**	**3**	4	4	5	7	9	11	13	15	16
	**4**	3	4	5	6	8	10	12	13	15
	**5**	3	3	4	5	7	9	11	12	13
	**6**	2	3	3	5	7	8	10	11	12
	**7**	2	2	3	4	6	8	9	10	11
	**8**	1	2	3	4	5	7	8	9	10
	**9**	1	2	2	3	5	6	8	9	10
	**10**	1	1	2	3	4	6	7	8	9
	**11**	1	1	1	2	4	5	7	7	8
	**12**	0	1	1	2	3	5	6	7	8
	**13**	0	1	1	2	3	4	6	6	7
	**14**	0	0	1	2	3	4	5	6	7
	**15**	0	0	1	1	2	4	5	5	6
	**16**	0	0	0	1	2	3	4	5	6
	**17**	0	0	0	1	2	3	4	4	5
	**18**	0	0	0	1	2	3	4	4	5
	**19**	0	0	0	1	1	2	3	4	5
	**20**	0	0	0	1	1	2	3	4	4

**Table 3:** Normative data for the number of mastication cycles

Gender	Age	Percentiles (number of mastication cycles)								
		2.5	5	10	25	50	75	90	95	97.5
**Male**	**3**	62.0	64.0	68.0	73.0	79.0	85.0	91.0	95.0	98.0
	**4**	58.0	60.0	63.0	69.0	75.0	81.0	86.0	90.0	93.0
	**5**	54.0	57.0	60.0	65.0	70.0	76.0	82.0	85.0	88.0
	**6**	51.0	53.0	56.0	61.0	66.0	72.0	77.0	80.0	83.0
	**7**	48.0	50.0	53.0	57.0	62.0	68.0	73.0	76.0	79.0
	**8**	44.0	47.0	49.0	54.0	59.0	64.0	69.0	72.0	75.0
	**9**	41.0	44.0	46.0	51.0	56.0	61.0	65.0	68.0	71.0
	**10**	39.0	41.0	43.0	48.0	52.0	57.0	62.0	65.0	67.0
	**11**	36.0	38.0	41.0	45.0	49.0	54.0	59.0	62.0	64.0
	**12**	34.0	36.0	38.0	42.0	47.0	51.0	56.0	58.0	61.0
	**13**	31.0	33.0	36.0	39.0	44.0	48.0	53.0	55.0	58.0
	**14**	29.0	31.0	33.0	37.0	41.0	46.0	50.0	53.0	55.0
	**15**	27.0	29.0	31.0	35.0	39.0	43.0	47.0	50.0	52.0
	**16**	25.0	27.0	29.0	33.0	37.0	41.0	45.0	48.0	50.0
	**17**	23.0	25.0	27.0	31.0	35.0	39.0	43.0	45.0	47.0
	**18**	22.0	23.0	25.0	29.0	33.0	37.0	41.0	43.0	45.0
	**19**	20.0	22.0	24.0	27.0	31.0	35.0	39.0	41.0	43.0
	**20**	19.0	20.0	22.0	25.0	29.0	33.0	37.0	39.0	41.0
**Female**	**3**	66.0	69.0	72.0	77.0	84.0	90.0	96.0	100.0	103.0
	**4**	62.0	65.0	68.0	73.0	79.0	85.0	91.0	95.0	98.0
	**5**	58.0	61.0	64.0	69.0	75.0	81.0	87.0	90.0	93.0
	**6**	55.0	57.0	60.0	65.0	71.0	77.0	82.0	85.0	88.0
	**7**	52.0	54.0	57.0	62.0	67.0	73.0	78.0	81.0	84.0
	**8**	48.0	51.0	53.0	58.0	64.0	69.0	74.0	77.0	80.0
	**9**	45.0	48.0	50.0	55.0	60.0	65.0	70.0	73.0	76.0
	**10**	43.0	45.0	47.0	52.0	57.0	62.0	67.0	70.0	72.0
	**11**	40.0	42.0	45.0	49.0	54.0	59.0	64.0	67.0	69.0
	**12**	37.0	40.0	42.0	46.0	51.0	56.0	61.0	63.0	66.0
	**13**	35.0	37.0	40.0	44.0	48.0	53.0	58.0	60.0	63.0
	**14**	33.0	35.0	37.0	41.0	46.0	50.0	55.0	58.0	60.0
	**15**	31.0	33.0	35.0	39.0	43.0	48.0	52.0	55.0	57.0
	**16**	29.0	31.0	33.0	37.0	41.0	45.0	47.0	52.0	54.0
	**17**	27.0	29.0	31.0	34.0	39.0	43.0	47.0	50.0	52.0
	**18**	25.0	27.0	29.0	33.0	37.0	41.0	45.0	47.0	50.0
	**19**	23.0	25.0	27.0	31.0	35.0	39.0	43.0	45.0	47.0
	**20**	22.0	24.0	26.0	29.0	33.0	37.0	41.0	43.0	45.0

**Table 4:** Normative data for the number of swallows

Gender	Age	Percentiles (number of swallows)								
		2.5	5	10	25	50	75	90	95	97.5
**Male**	**3**	0.0	1.0	1.0	2.0	4.0	5.0	6.0	7.0	8.0
	**4**	0.0	1.0	1.0	2.0	4.0	5.0	6.0	7.0	8.0
	**5**	0.0	1.0	1.0	2.0	3.0	5.0	6.0	7.0	8.0
	**6**	0.0	1.0	1.0	2.0	3.0	5.0	6.0	7.0	8.0
	**7**	0.0	1.0	1.0	2.0	3.0	5.0	6.0	7.0	8.0
	**8**	0.0	1.0	1.0	2.0	3.0	4.0	6.0	7.0	7.0
	**9**	0.0	1.0	1.0	2.0	3.0	4.0	6.0	7.0	7.0
	**10**	0.0	1.0	1.0	2.0	3.0	4.0	6.0	6.0	7.0
	**11**	0.0	1.0	1.0	2.0	3.0	4.0	5.0	6.0	7.0
	**12**	0.0	1.0	1.0	2.0	3.0	4.0	5.0	6.0	7.0
	**13**	0.0	0.0	1.0	2.0	3.0	4.0	5.0	6.0	7.0
	**14**	0.0	0.0	1.0	2.0	3.0	4.0	5.0	6.0	7.0
	**15**	0.0	0.0	1.0	2.0	3.0	4.0	5.0	6.0	7.0
	**16**	0.0	0.0	1.0	2.0	3.0	4.0	5.0	6.0	7.0
	**17**	0.0	0.0	1.0	2.0	3.0	4.0	5.0	6.0	7.0
	**18**	0.0	0.0	1.0	1.0	2.0	4.0	5.0	6.0	6.0
	**19**	0.0	0.0	1.0	1.0	2.0	4.0	5.0	6.0	6.0
	**20**	0.0	0.0	1.0	1.0	2.0	4.0	5.0	6.0	6.0
**Female**	**3**	1.0	1.0	1.0	2.0	4.0	5.0	7.0	8.0	8.0
	**4**	1.0	1.0	1.0	2.0	4.0	5.0	6.0	7.0	8.0
	**5**	0.0	1.0	1.0	2.0	3.0	5.0	6.0	7.0	8.0
	**6**	0.0	1.0	1.0	2.0	3.0	5.0	6.0	7.0	8.0
	**7**	0.0	1.0	1.0	2.0	3.0	4.0	6.0	7.0	8.0
	**8**	0.0	1.0	1.0	2.0	3.0	4.0	6.0	6.0	7.0
	**9**	0.0	1.0	1.0	2.0	3.0	4.0	6.0	6.0	7.0
	**10**	0.0	1.0	1.0	2.0	3.0	4.0	6.0	6.0	7.0
	**11**	0.0	0.0	1.0	2.0	3.0	4.0	5.0	6.0	6.0
	**12**	0.0	0.0	1.0	1.0	2.0	4.0	5.0	6.0	6.0
	**13**	0.0	0.0	1.0	1.0	2.0	4.0	5.0	5.0	6.0
	**14**	0.0	0.0	1.0	1.0	2.0	3.0	5.0	5.0	6.0
	**15**	0.0	0.0	0.0	1.0	2.0	3.0	4.0	5.0	6.0
	**16**	0.0	0.0	0.0	1.0	2.0	3.0	4.0	5.0	6.0
	**17**	0.0	0.0	0.0	1.0	2.0	3.0	4.0	5.0	6.0
	**18**	0.0	0.0	0.0	1.0	2.0	3.0	4.0	5.0	5.0
	**19**	0.0	0.0	0.0	1.0	2.0	3.0	4.0	5.0	5.0
	**20**	0.0	0.0	0.0	1.0	2.0	3.0	4.0	5.0	5.0

**Table 5:** Normative data for the ToMaSS-C test duration in seconds

Gender	Age	Percentiles (ToMaSS test duration in seconds)								
		2.5	5	10	25	50	75	90	95	97.5
**Male**	**3**	33.4	36.5	40.5	48.0	58.1	70.2	83.4	92.4	101.0
	**4**	31.8	34.7	38.4	45.6	55.0	66.5	78.8	87.2	95.3
	**5**	30.1	32.9	36.4	43.2	52.1	62.9	74.6	82.6	90.2
	**6**	28.6	31.2	34.6	40.9	49.4	59.6	70.6	78.0	85.1
	**7**	27.2	29.6	32.8	38.8	46.7	56.4	66.7	73.8	80.6
	**8**	25.7	28.1	31.0	36.7	44.3	53.4	63.1	69.8	76.2
	**9**	24.4	26.6	29.5	34.8	42.0	50.6	59.8	66.2	72.2
	**10**	23.1	25.2	27.9	33.0	39.7	47.9	56.7	62.7	68.4
	**11**	21.8	23.9	26.4	31.2	37.6	45.4	53.7	59.4	64.8
	**12**	20.7	22.6	25.0	29.6	35.7	43.0	50.9	56.3	61.4
	**13**	19.6	21.4	23.7	28.0	33.8	40.7	48.2	53.4	58.3
	**14**	18.5	20.2	22.4	26.5	32.0	38.6	45.7	50.6	55.3
	**15**	17.5	19.1	21.2	25.1	30.3	36.6	43.4	48.0	52.5
	**16**	16.5	18.1	20.0	23.7	28.7	34.7	41.2	45.6	49.8
	**17**	15.6	17.1	18.9	22.5	27.2	32.9	39.0	43.3	47.3
	**18**	14.7	16.1	17.9	21.2	25.7	31.2	37.1	41.1	45.0
	**19**	13.9	15.2	16.9	20.1	24.4	29.6	35.2	39.1	42.8
	**20**	13.1	14.3	16.0	19.0	23.1	28.1	33.5	37.2	40.7
**Female**	**3**	37.4	40.9	45.3	53.7	64.9	78.5	93.1	103.1	112.6
	**4**	35.4	38.6	42.7	50.7	61.2	73.9	87.6	97.0	105.9
	**5**	33.4	36.4	40.3	47.7	57.6	69.6	82.5	91.2	99.7
	**6**	31.5	34.4	38.0	45.0	54.3	65.5	77.4	85.7	93.6
	**7**	29.7	32.4	35.8	42.4	51.1	61.6	72.9	80.7	88.0
	**8**	28.0	30.5	33.8	39.9	48.2	58.1	68.6	75.9	82.9
	**9**	26.3	28.8	31.8	37.6	45.4	54.7	64.7	71.6	78.2
	**10**	24.9	27.1	30.0	35.4	42.7	51.5	60.9	67.4	73.6
	**11**	23.4	25.5	28.2	33.4	40.3	48.6	57.5	63.6	69.4
	**12**	22.0	24.0	26.6	31.4	37.9	45.7	54.1	59.8	65.3
	**13**	20.7	22.6	25.0	29.6	35.7	43.1	51.0	56.4	61.6
	**14**	19.5	21.3	23.5	27.9	33.6	40.6	48.1	53.2	58.1
	**15**	18.3	20.0	22.1	26.3	31.7	38.3	45.3	50.2	54.8
	**16**	17.2	18.8	20.8	24.7	29.9	36.1	42.8	47.4	51.8
	**17**	16.2	17.7	19.6	23.3	28.1	34.0	40.4	44.8	48.9
	**18**	15.2	16.6	18.4	21.9	26.5	32.0	38.1	42.2	46.2
	**19**	14.3	15.6	17.3	20.6	25.0	30.3	36.0	39.9	43.7
	**20**	13.4	14.6	16.2	19.4	23.5	28.5	34.0	37.8	41.3

Model comparisons with age fitted as a categorical variable (e.g., chews AICs = 1114.2) and then as a continuous variable (e.g., chews AICs = 1158.6) showed that age should be used as a continuous variable in further statistical analysis rather than grouped.

No statistically significant interaction effect of age and gender was found for any of the response variables (Supplementary material 1). This implies that age affects boys and girls in the same way. Age was found to have a statistically significant effect on all the outputs: (${\chi }^{2}$(2) = 104.710, *p* < 0.001), (${\chi }^{2}$(2) = 375.890, *p* < 0.001), (${\chi }^{2}$(2) = 8.537, *p* = 0.014) and ($\mathrm{F}$(2,110) = 52.226, *p* < 0.001) for bites, chews, swallows and time, respectively, whereas gender significantly affected only bites (${\chi }^{2}$(2) = 11.527, *p* = 0.003) and chews (${\chi }^{2}$(2) = 10.300, *p* = 0.006).

Figure S1 in Supplementary material 2 shows estimated mean gender-specific age-related trends for ToMaSS-C parameters. While the overlapping 95% envelopes show a lack of gender-specific differences, the gaps between the envelopes for expected bites and masticatory cycles suggest a statistically significant difference for 7–12 aged females. It was estimated that, on average, they make more bites and masticatory cycles than males around the ages of 7 to 12 years.

Equivalence testing found no equivalence between the ToMaSS-C datasets that were collected manually (historical data) and through the application. The 90% CIs for the logged mean ratio lay completely outside the pre-set equivalent bounds (Δ = 20%) for all ToMaSS-C parameters (Supplementary material 3).

## Discussion

The current piloting project aimed to establish a preliminary normative dataset for the ToMaSSApp™ in children (4 to 19 years old) for the Arnott’s Salada™ original cracker and compare that with the historical data collected manually.

### ToMaSSApp™ age and gender effects

Age was found to have a statistically significant effect on all oropharyngeal efficiency measures registered using ToMaSSApp™. Even though age was fitted as a continuous rather than categorical variable, results of the current study are in line with the findings of Frank et al. [17] and indicate that efficiency of solids consumption is impacted by developmental processes, with age affecting both neurodevelopmental [24, 25] and anatomical maturation [15, 26–28]. Inclusion of 18- and 19-year-old participants allowed creation of normative tables for people aged 3 to 20 years.

While the age affected both genders equally - i.e., no statistically significant interactions were found between gender and age - the results showed that females, on average, took more bites and masticatory cycles to consume the cracker than males did. This finding aligns with the past paediatric [17, 18] and adult research [10, 29–34] and may be explained by different developmental processes for bites and mastications across genders, as found in Almotairy et al.’s systematic review [15]. These earlier studies showed gender differences in bite force, jaw kinematics, and chewing efficiency. Barrera et al. [26] also found significant differences in the rates of change for the median particle size of food across genders. The researchers attributed this to the difference in dentition eruption. Additionally, earlier permanent dental eruption in girls [35–37] could explain a gap between genders for bites and chews around 7 to 12 years of age (Supplementary material 2). Future paediatric study designs should account not only for the dentition change (primary vs. permanent) but also for the dental health that significantly impacts chewing performance [38–40].

### ToMaSSApp™ vs. manual ToMaSS-C administration

Equivalence testing of two normative datasets (historical data obtained manually and current data obtained using the ToMaSSApp™) revealed no equivalence. The most prominent difference is observed in the youngest age groups (Supplementary material 3), making more bites and, especially, masticatory cycles registered using the ToMaSSApp™. In those younger age groups, females showed a more prominent difference than boys, which again could be explained by the earlier dentition eruption and maturation discussed in the paragraph above. Absence of equivalence between two datasets may suggest that manual administration may be more challenging, as the observer might miss the bites and masticatory cycles while taking written notes. This issue may be more likely to occur in the younger age groups, who exhibited more frequent bites and masticatory cycles in the current study and engaged in more body movements during testing, as reported in Porter et al. [18]. This finding should be confirmed in future equivalence studies with a larger sample size and supported by the reliability assessments discussed in the following paragraph. A larger study could result in narrower confidence intervals, which may allow the establishment of equivalence between the two datasets.

### Limitations and considerations

The main limitation of this pilot study is that reliability testing was not incorporated in its design. A formal reliability study was planned as the next step, given that ToMaSSApp™ is applicable in the paediatric population. Future research should include repeated on-site assessments by two independent raters and repeated evaluation of video-recorded performances using both manual and app-based ToMaSS-C methods to establish test–retest, inter-rater, and intra-rater reliability and equivalence within the same cohort. If the absence of equivalence is confirmed, further investigation would be required to determine whether discrepancies are attributable to the app-based recording method, the manual recording method, or both. Depending on these findings, development of a separate, larger paediatric normative dataset for the ToMaSSApp™ could be needed. If the two methods show equivalent results, the normative data may be used as a reference with either the ToMaSSApp™ or the manual recording approach.

The next limitation of the current study is that younger children did not always follow the instruction to repeat their name after finishing the cracker. In these instances, the researcher had to promptly encourage the children to say their name or confirm they had finished. This prompt may have extended the ToMaSS-C duration. Therefore, an adapted instruction for the younger children may be necessary, alongside further identification of which ages benefit most from that adaptation. For example, asking children to say “ah” after they had finished their cracker has been successfully used in research involving children aged 2 to 3 years [18]. However, In’t Veld et al. [19] asserted that the language comprehension skills of typical 3-year-olds are sufficient to follow the original ToMaSS instructions. Another possible explanation is that, although children with reported speech or language difficulties were excluded based on parent- or self-report screening, no formal language assessment was conducted. Therefore, undiagnosed or subtle receptive language difficulties may have influenced some children’s ability to follow the task instructions. Future research may further explore the interaction between age-related language development and ToMaSS-C task compliance.

The environment, along with varying physical states and hunger levels among children, may have also influenced the ToMaSS-C values. Since the data collection occurred in public spaces, many children were observed by multiple individuals (e.g., their mates) during the testing, and some participants attempted to exaggerate their cracker consumption. While eating often happens in a social setting, being watched by friends is quite different from the controlled environment of a clinical setting where swallowing performance is typically assessed. Further, a large majority of the children participated in the study after playing sports, which may affect saliva composition and secretion [41] and impact hunger [42]. Saliva production has been shown to be influenced by hunger intensity [43] and has the predictive ability of satiation [44]. Therefore, differences or malfunctions (e.g., xerostomia) in saliva production may impact chewing and swallowing efficiency and subsequently ToMaSS results.

The test requirement to eat the wheat-containing Arnott’s Salada™ excludes those who were on a gluten-free diet. Considering the increasing prevalence of coeliac disease diagnoses [45], future research should consider establishing normative data for the ToMaSS-C using a gluten-free cracker.

Finally, while the validity of the ToMaSS has been confirmed in adults using other dysphagia-specific tools and instrumental imaging studies [13, 14, 46], similar studies are needed for the paediatric population.

### Clinical implications

By today, the ToMaSS-C assessment has been shown to identify impaired chewing and swallowing efficiency in children with Down syndrome [19] and OMD [20], while ToMaSSApp™ can improve the administration of the assessment. This improvement may be particularly notable when working with younger children, as shown in the current research’s findings. Having normative data that has been created through ToMaSSApp™ could provide clinicians with norms to judge, develop a management plan (e.g., inclusion of chewing exercises) and follow up on children’s ability to chew and swallow solids.

Examples of populations that may benefit from the use of ToMaSS-C and the established normative data are children with OMD, dysphagia, and CP. The CP is a nonprogressive movement disorder that can negatively influence oral motor skills [47]. In contrast to the available KCPS [7], MAT [8] and paediatric feeding assessment tools evaluating broader feeding domains [5], ToMaSS is an objective test, which specifically focuses on quantifiable measures of masticatory performance and requires a minimum technology support—a smartphone with the installed ToMaSSApp™. Such metrics may support more precise identification of masticatory inefficiency, facilitate follow-up on changes, and assist in evaluating intervention outcomes. Future studies may investigate ToMaSS-C’s ability to detect changes in masticatory skills in children with CP undergoing chewing training, which has been shown to be effective in this population [48].

## Conclusion

The current study established a preliminary paediatric normative dataset for the ToMaSS-C through ToMaSSApp™ administration in 114 healthy children. The results from the current research project replicate those of previous studies concerning ToMaSS-C in the paediatric population. ToMaSSApp™ is thus shown to be a feasible and efficient tool for administering ToMaSS-C. Establishing normative data for ToMaSS-C is essential for understanding the typical processes of oropharyngeal efficiency as well as identifying diagnostic and management pathways and the effectiveness of interventions targeting solid consumption skills. The data collated in this study provides a beneficial foundation for developing the normative dataset. Additional research with a larger sample size is needed to confirm the validity of ToMaSS-C in children (e.g., comparing to the Videofluoroscopy Swallow Study), along with an investigation into year-by-year age stratification and age-appropriate instructions in clinical settings.

## Supplementary Information

Below is the link to the electronic supplementary material.

ESM 1 (DOCX 17.0 KB)

ESM 2 (DOCX 221 KB)

ESM 3 (DOCX 93.3 KB)

## Data Availability

No datasets were generated or analysed during the current study.

## Abbreviation

CP: Cerebral palsy
KCPS: Karaduman Chewing Performance Scale
MAT: Mixing Ability Test
OMD: Oral motor disorders
ToMaSS: Test of Masticating and Swallowing Solids
ToMaSSApp: Test of Masticating and Swallowing Solids Application
