# Supplementary material for: Establishing a preliminary normative database of oral efficiency for children: the Test of Masticating and Swallowing Solids Application (ToMaSSApp)
Source: Eur J Pediatr. 2026 Jun 3;185(7):463. doi: 10.1007/s00431-026-07110-2 (PMC13234021; doi:10.1007/s00431-026-07110-2)
Supplement: Supplementary file 1 — (DOCX 17.0 KB) [file 431_2026_7110_MOESM1_ESM.docx]

**Supplementary material 1**

**Table S1. Effects of age and gender on TOMASS parameters**

|  | **TOMASS parameters** | | | |
| --- | --- | --- | --- | --- |
|  | **Bites** | **Masticatory cycles** | **Swallows** | **Time** |
| **Gender** | $\chi^{2}$(2) = 11.527,  *p* = .003 | $\chi^{2}$(2) = 10.300,  *p* = .006 | $\chi^{2}$(2) = 1.337,  *p* = .512 | F(2, 110) = 1.165,  *p* = .316 |
| **Age** | $\chi^{2}$(2) = 104.710,  *p* < .001 | $\chi^{2}$(2) = 375.890,  *p* < .001 | $\chi^{2}$(2) = 8.537,  *p* = .014 | F(2, 110) = 52.226,  *p* < .001 |
| **Interaction** | $\chi^{2}$(1) = 0.987,  *p* = .320 | $\chi^{2}$(1) = 0.412,  *p* = .521 | $\chi^{2}$(1) = 0.753,  *p* = .386 | F(1, 110) = 0.244,  *p* = .622 |

Two-way ANOVAs, model comparison was conducted using likelihood ratio *χ²*-tests for generalized linear models and *F*-tests for linear models (for Time).
