# Supplementary material for: Establishing a preliminary normative database of oral efficiency for children: the Test of Masticating and Swallowing Solids Application (ToMaSSApp)
Source: Eur J Pediatr. 2026 Jun 3;185(7):463. doi: 10.1007/s00431-026-07110-2 (PMC13234021; doi:10.1007/s00431-026-07110-2)
Supplement: Supplementary file 2 — (DOCX 221 KB) [file 431_2026_7110_MOESM2_ESM.docx]

**Supplementary material 2**

**Table S1. Estimated changes (% decrease per year of age) of ToMaSS parameters with age by gender**

| **ToMaSS parameter** | **Female** | | **Male** | |
| --- | --- | --- | --- | --- |
|  | **%-decrease**  **(95% CI)** | ***P*-value** | **%-decrease**  **(95% CI)** | ***P*-value** |
| **Bites** | 11.5  (8.0, 15.2) | <0.001 | 14.5  (9.9, 19.3) | <0.001 |
| **Mastication cycles** | 5.6  (4.8, 6.5) | <0.001 | 6.1  (5.1, 7.0) | <0.001 |
| **Swallows** | 4.6  (1.0, 8.3) | 0.013 | 2.3  (-1.1, 5.9) | 0.184 |
| **Time** | 6.2  (4.6, 7.8) | <0.001 | 5.6  (3.9, 7.3) | <0.001 |

**Fig. S1 Estimated mean age trends and their 95% confidence intervals of ToMaSS parameters**Overlapping confidence envelopes for females and males indicate no statistically significant differences between genders. The gaps between the intervals for expected bites and masticatory cycles suggest a statistically significant difference, with females, on average, making more bites and masticatory cycles than males around the ages of 7 to 12 years.
