# Supplementary material for: Establishing a preliminary normative database of oral efficiency for children: the Test of Masticating and Swallowing Solids Application (ToMaSSApp)
Source: Eur J Pediatr. 2026 Jun 3;185(7):463. doi: 10.1007/s00431-026-07110-2 (PMC13234021; doi:10.1007/s00431-026-07110-2)
Supplement: Supplementary file 3 — (DOCX 93.3 KB) [file 431_2026_7110_MOESM3_ESM.docx]

**Supplementary material 3. Equivalence testing: Comparison between ToMaSS-C datasets collected via the application and manually**

**Fig S1. Logged ratio of the estimated mean number of bites for the app and manual sources**


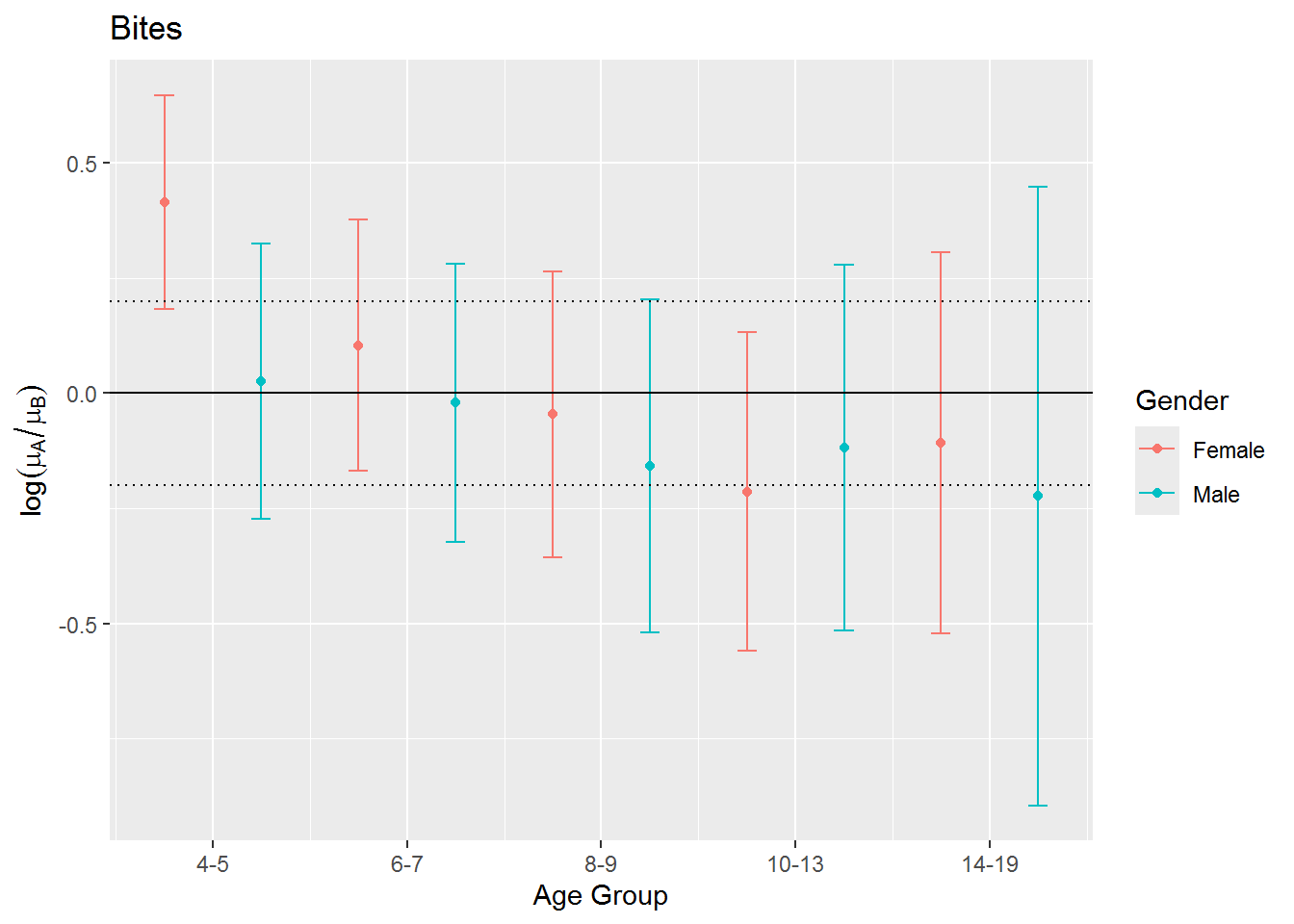


The equivalence can be concluded if all the confidence intervals fall entirely within the pre-set practically equivalent bounds. In the figure above, the limits for Δ = 20% are shown. Equivalence can only be concluded for Δ ≥ 144.6 %.

**Fig S2. Logged ratio of the estimated mean number of mastication cycles for the app and manual sources**


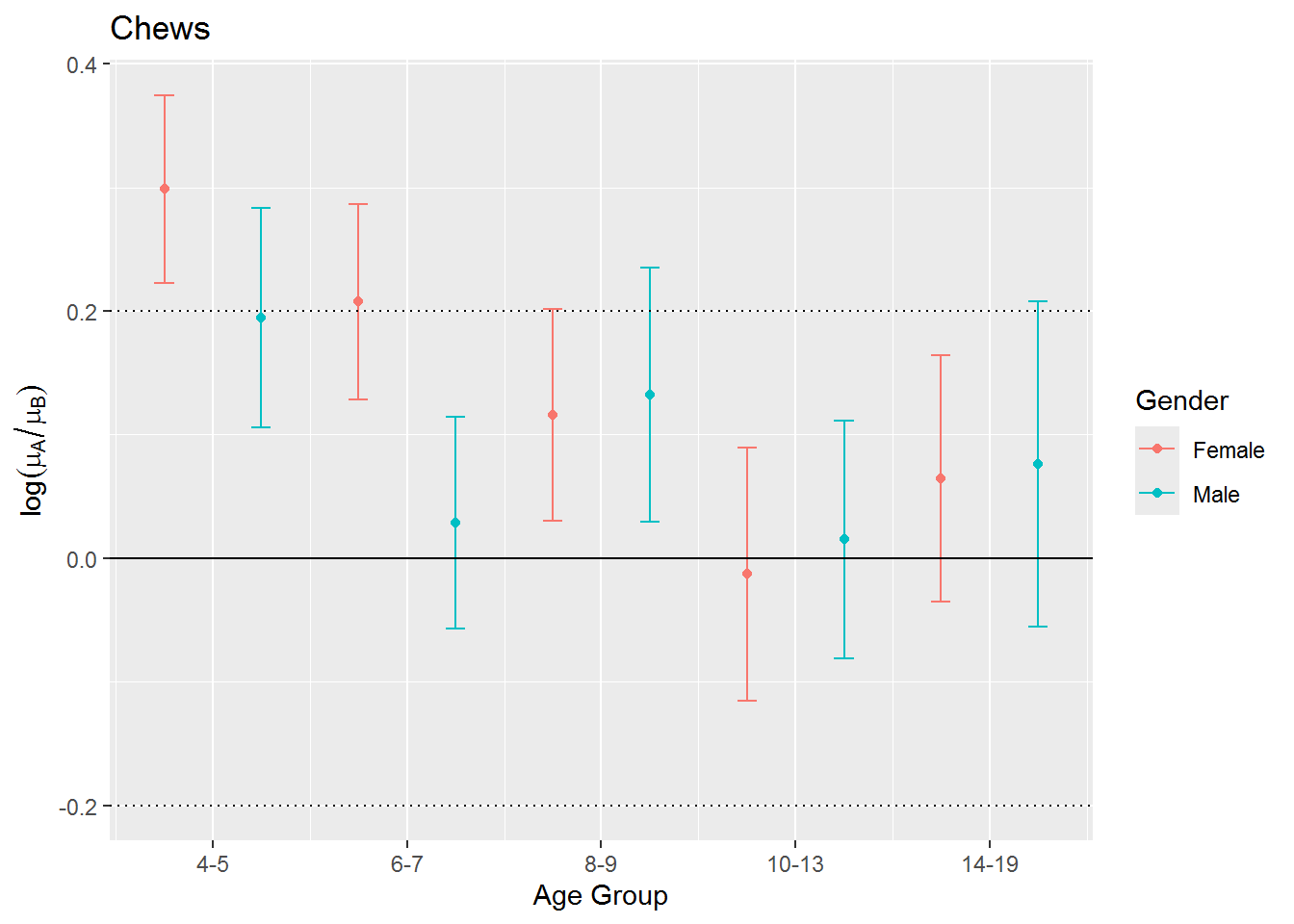


The equivalence can be concluded if all the confidence intervals fall entirely within the pre-set practically equivalent bounds. In the figure above, the limits for Δ = 20% are shown. Equivalence can only be concluded for Δ ≥ 45.5 %.

**Fig S3. Logged ratio of the estimated mean number of swallows for the app and manual sources**


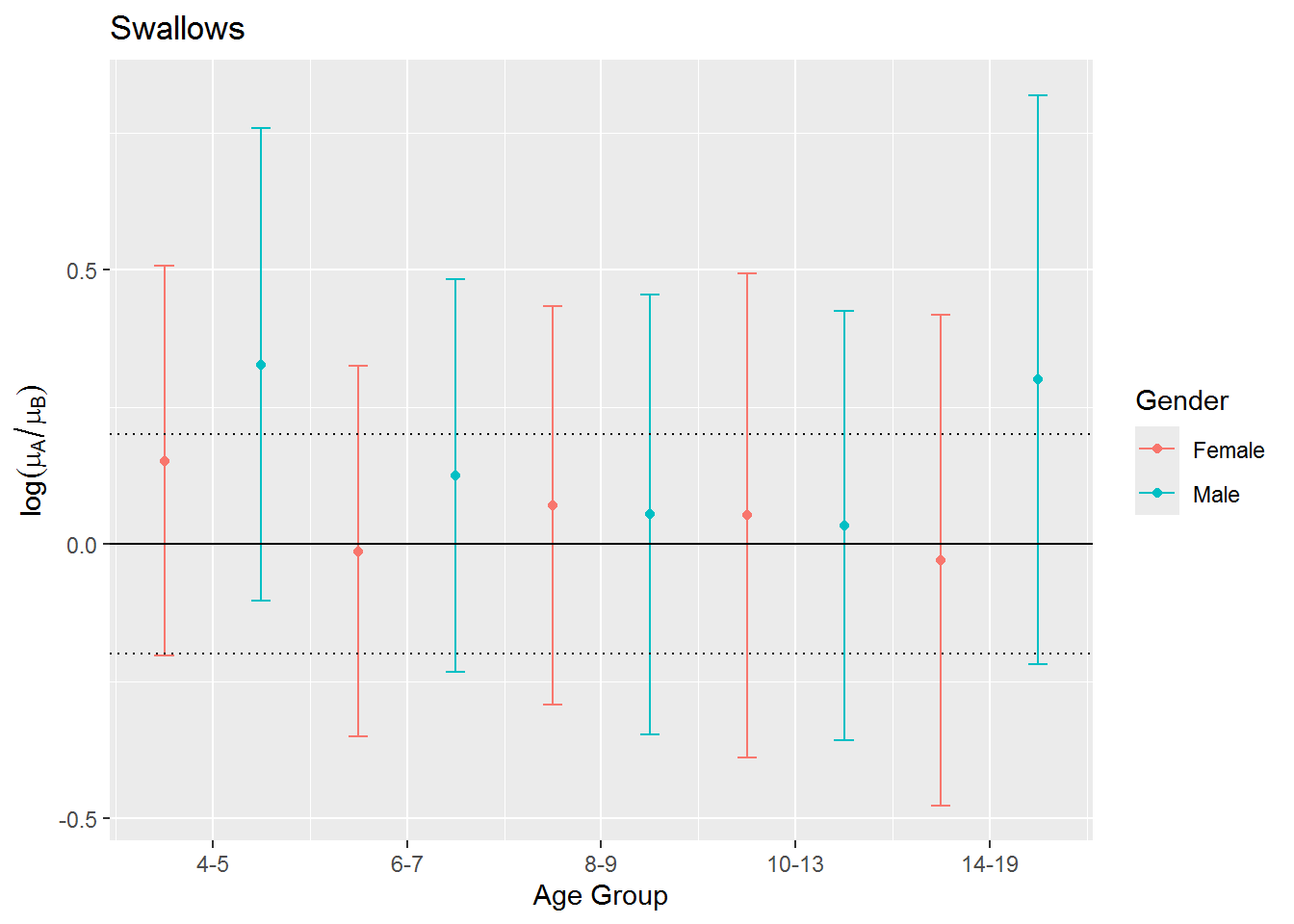


The equivalence can be concluded if all the confidence intervals fall entirely within the pre-set practically equivalent bounds. In the figure above, the limits for Δ = 20% are shown. Equivalence can only be concluded for Δ ≥ 126.8 %.

**Fig S4. Logged ratio of the estimated mean number of swallows for the app and manual sources**


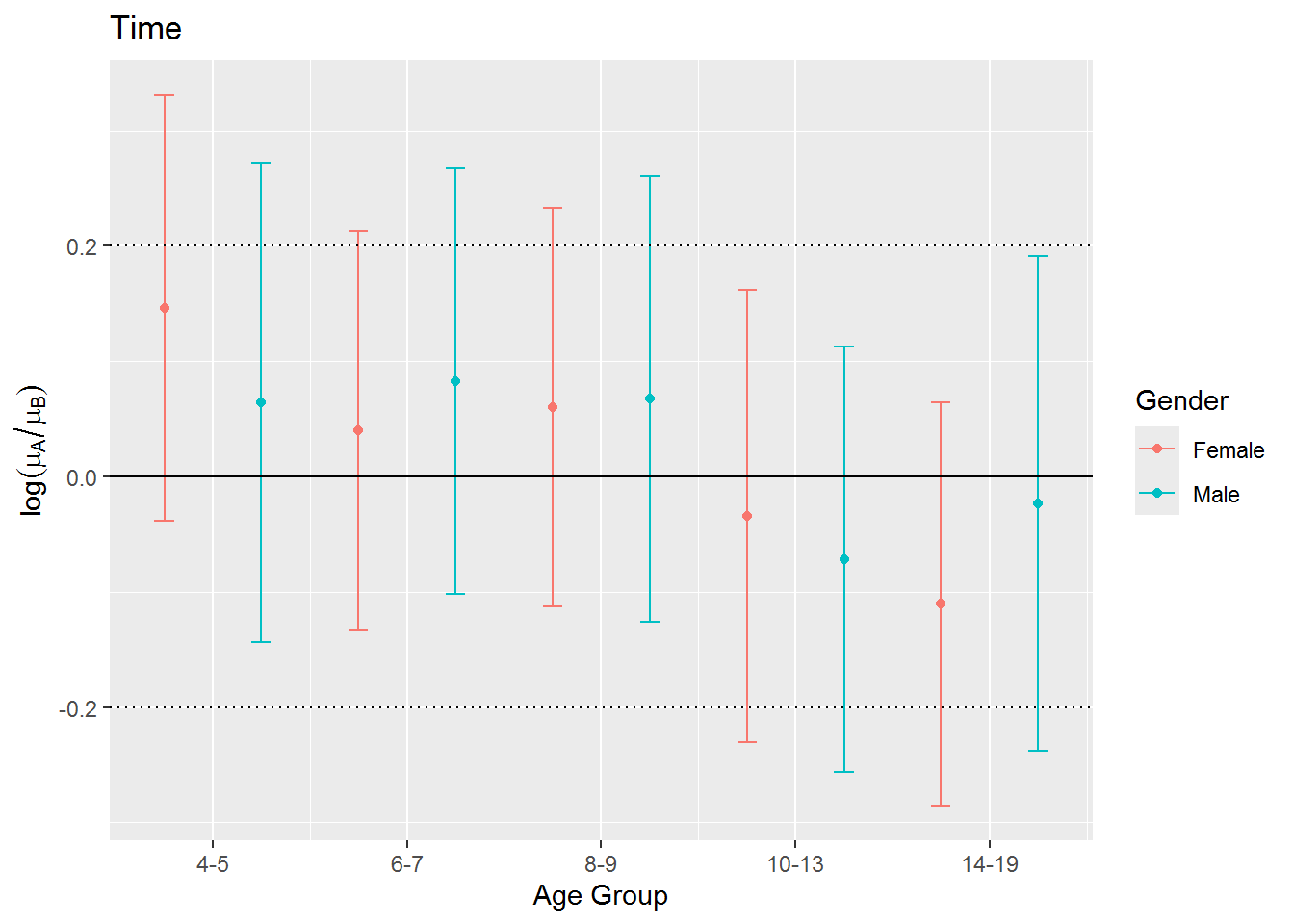


The equivalence can be concluded if all the confidence intervals fall entirely within the pre-set practically equivalent bounds. In the figure above, the limits for Δ = 20% are shown. Equivalence can only be concluded for Δ ≥ 39.2 %.
